# Seropositivity of COVID-19 among asymptomatic healthcare workers: A multi-site prospective cohort study from Northern Virginia, United States

**DOI:** 10.1016/j.lana.2021.100030

**Published:** 2021-07-29

**Authors:** Abdulla A. Damluji, Siqi Wei, Scott A. Bruce, Amanda Haymond, Emanuel F. Petricoin, Lance Liotta, G. Larry Maxwell, Brian C Moore, Rachel Bell, Stephanie Garofalo, Eric R Houpt, David Trump, Christopher R. deFilippi

**Affiliations:** a The Inova Center of Outcomes Research, Inova Heart and Vascular Institute, 3300 Gallows Road, I-465, Falls Church, VA 22042, United States; b Division of Cardiology, Johns Hopkins University School of Medicine, Baltimore, MD, United States; c Department of Statistics, George Mason University, Fairfax, VA, United States; d Department of Statistics, Texas A&M University, College Station, TX, United States; e Center for Applied Proteomics and Molecular Medicine, George Mason University, Manassas, VA, United States; f Division of Infectious Diseases and International Health, University of Virginia, Charlottesville, VA, United States; g Virginia Department of Health, Richmond, VA, United States

**Keywords:** COVID-19, Immunity, Serologic test, CIA, Chemiluminescent immunoassay, COVID-19, Coronavirus disease 2019, HCW, Healthcare worker, PPE, Personal protective equipment, SARS-CoV-2, severe acute respiratory syndrome coronavirus 2, ZIP, Zone Improvement Plan

## Abstract

**Background:**

Because of their direct patient contact, healthcare workers (HCW) face an unprecedented risk of exposure to COVID-19. The aim of this study was to examine incidence of COVID-19 disease among asymptomatic HCW and community participants in Northern Virginia during 6 months of follow-up.

**Methods:**

This is a prospective cohort study that enrolled healthy HCW and residents who never had a symptomatic COVID-19 infection prior to enrolment from the community in Northern Virginia from April to November 2020. All participants were invited to enrol in study, and they were followed at 2-, and 6-months intervals. Participants were evaluated by commercial chemiluminescence SARS-CoV-2 serology assays as part of regional health system and public health surveillance program to monitor the spread of COVID-19 disease.

**Findings:**

Of a total of 1,819 asymptomatic HCW enrolled, 1,473 (96%) had data at two-months interval, and 1,323 (73%) participants had data at 6-months interval. At baseline, 21 (1.15%) were found to have prior COVID-19 exposure. At two-months interval, COVID-19 rate was 2.8% and at six months follow-up, the overall incidence rate increased to 4.8%, but was as high as 7.9% among those who belong to the youngest age group (20–29 years). Seroconversion rates in HCW were comparable to the seropositive rates in the Northern Virginia community. The overall incidence of COVID-19 in the community was 4.5%, but the estimate was higher among Hispanic ethnicity (incidence rate = 15.3%) potentially reflecting different socio-economic factors among the community participants and the HCW group. Using cross-sectional logistic regression and spatio-temporal mixed effects models, significant factors that influence the transmission rate among HCW include age, race/ethnicity, resident ZIP-code, and household exposure, but not direct patient contact.

**Interpretation:**

In Northern Virginia, the seropositive rate of COVID-19 disease among HCW was comparable to that in the community.

**Research in context**

**Evidence before this study**

• Healthcare workers (HCWs) experience significant burden from COVID-19 disease because of their direct patient care with infected individuals.

**Added value of this study**

• The overall prevalence of COVID-19 exposure reflected by serology testing in asymptomatic HCW at baseline in April to May 2020 near the beginning of the pandemic was low.
• With implementation of public health measures and PPE in hospital settings the overall incidence rate of SARS-CoV-2 exposure reflected by incident serology among HCW over the next 6 months of the pandemic remained low (~ 4.8%).
• The incidence rate of COVID-19 disease among HCW is similar to the incidence rate of the disease among asymptomatic participants from the community with the important exception of Hispanic ethnicity potentially reflecting different socio-economic factors among those Hispanics living in the community and the subset employed in the regional healthcare system.
• The main factors that influence the incidence rate among HCW are younger age groups, race, residential zip code, and exposure to a COVID-19 infected individual within the household and not factors such as direct patient contact or work location within the healthcare system

**Implications of all available evidence**

• In Northern Virginia, the seropositive rate of COVID-19 disease among HCW was comparable to that in the community at large.

Alt-text: Unlabelled box

## Introduction

In March 2020, the World Health Organization declared the coronavirus disease 2019 (COVID-19) a pandemic with millions of people infected worldwide [1]. The disease is caused by severe acute respiratory syndrome coronavirus 2 (SARS-CoV-2) that belongs to the beta coronavirus genus [2]. This virus is capable of human-to-human transmission and spreads via respiratory droplets causing a respiratory illness that closely resembles SARS-CoV infection [2]. The aggressive inflammatory response to COVID-19 can result in airway damage, respiratory failure, cardiac injury, and multiorgan failure, which lead to death in susceptible patients [3].

Healthcare workers (HCW) faced an unprecedented risk of exposure to SARS-CoV-2 because of their direct patient contact. While some develop moderate to severe disease, most become infected with no or mild symptoms [4]. Overall, an estimated 40 to 45% of infected individuals may be asymptomatic or mildly symptomatic [5]. Because HCW are more likely to be studied when compared to community based participants this results in potential selection bias rather than different clinical case presentation between the two groups [6,7]. In high-exposure settings, the ability to accurately assess the prevalence and seropositive rate of infection among HCW has been challenging as many estimates are derived from small size cohorts or cross-sectional studies without longitudinal follow-up [4]. The aims of this study are to: (1) identify the initial prevalence of SARS-CoV-2 exposure among healthcare professionals early in the pandemic, with and without direct patient contact, and other factors that can explain the prevalence of the disease among HCW; (2) examine the rate of seroconversion among asymptomatic HCW over time; (3) determine the factors that influence the seropositivity among asymptomatic HCW, as compared to asymptomatic members of the regional community selected for a public health serologic surveillance program.

## Methods

### Healthcare workers study population

This is a prospective cohort study that enrolled asymptomatic HCW in Northern Virginia, United States from April to November 2020, as part of a health surveillance program to monitor the spread of COVID-19 disease in a large healthcare system in the mid-Atlantic region. Asymptomatic HCW were included if they were >18 years of age, employed or contracted by a large integrated health system in Northern Virginia (Inova Health), and willing to sign an informed consent to participate and follow study procedures. Concerted efforts were made to enrol members of the ancillary staff, contracting, and other non-medical services to have a global representation of healthcare workforce within the system. Participants were excluded if there was a known or suspected symptomatic COVID-19 infection at first enrolment time point with symptoms including fever, cough or shortness of breath, but participants who developed symptoms after enrolment were continued to be followed in the study. After explaining the risks and benefits of enrolment, each participant was asked to read an electronic informed consent that explicitly defined the aims of this research study, duration of follow-up, all possible outcomes, the risks involved, and alternatives to participation. Each enrolled participant signed the electronic consent form using a signature pad and the document was saved to the electronic medical system. The protocol was approved by the Inova institutional review board. Healthcare structures included hospitals, outpatient clinics, community care clinics, within Inova Health, which is located in the Commonwealth of Virginia, United States.

### Community sample from Northern Virginia

Similar to the HCW cohort, prospective asymptomatic participants without a known COVID-19 diagnosis residing in the community were enrolled as part of the public health surveillance program in Virginia from June 1 to August 14, 2020 from 5 geographically diverse health system sites: the University of Virginia Health System, Inova Health System, Sentara, Healthcare, Carilion Clinic, and Virginia Commonwealth University [8]. Selection of the sites was based on geographically diverse health system sites from Northern, Eastern, and Western regions of the state of Virginia. All community participants had to go to one ambulatory clinic for testing. All adult outpatient participants presented in person for scheduled outpatient clinic or outpatient laboratory appointment were eligible to be enrolled in the community study [8]. All outpatient sites conducted pre-screening to ensure that participants did not have COVID-19 like symptoms prior to enrolment in the study. Participants were included in this analysis if they were adults >18 years of age, resided in the Northern Virginia region willing to sign an informed consent to participate in the study. The community participants were evaluated in a state wide cross-sectional surveillance program [8]. The use of community population was generalizable since it had demographics that matched the region and drew on multiple ambulatory clinics from a variety of different socio-economic districts within Northern Virginia.This protocol was approved by the University of Virginia institutional review board with a waiver of informed consent because the study was requested by the Virginia Department of Health as a public health surveillance according to 45 CFR §46.102. A full description of this cohort was previously published [8].

### Blood collection and laboratory assay measurements

All blood tests for the community cohort were performed at one site (University of Virginia Medical Laboratory, Charlottesville, VA) and plasma (lithium heparin tubes) was tested on the Architect i12000 analyser (Abbott) using SARS-CoV-2 immunoglobulin G antibody immunoassay for quality control [8]. Approximately 20 mL of blood was drawn at baseline, 2-month, and 6-months from each HCW participant. At each interval, the following was performed: COVID-19 IgM–IgG serologic testing to the spike protein at the Inova Health System Biocore laboratory, Falls Church, VA (Anti-SARS-CoV-2 Total Reagent Pack, Ortho Diagnostics, Raritan, NJ. A positive serology result was defined by 1.00 signal to calibrator (S/C) ratio as defined by the manufacturer [9]. Blood was stored for additional non-genetic tests for five years following enrolment. For northern Virginia community participants SARS-CoV-2 enrolment took place at one of several ambulatory clinics in June-July of 2020 and serology was testing with the Abbott Laboratories AdviseDx SARS-CoV-2 assay measured at the University of Virginia as described elsewhere [8]. The Abbott assay used was the anti-IgG II assay. The sensitivity and specificity for each of the assays were previously published [10].

### Clinical characteristics

For Inova HCW at baseline, the following data were collected: demographics, cardiovascular risk factors, Zone Improvement Plan (ZIP) code, and job description identified as “direct patient care” or “non-direct care”. Race and ethnicity are self-identified categories chosen by research participants. Home exposure was defined as a HCW with other domiciliary members with PCR confirmed infection. Direct patient care was defined as any person who works in a care provider capacity who comes within six feet of the patient. At the six months visit, HCW were also queried if they had participated in a vaccine study or if another household member had been diagnosed with COVID-19. During baseline, 2-, and 6-month visits, participants were screened for temperature and symptoms related to COVID-19 disease. The primary domiciliary ZIP code for each participant was collected.

### Descriptive statistics and cross-sectional analysis

During the study period, research participants were categorized into two distinct and separate categories: (1) Inova HCW and (2) community sample from Northern Virginia. The demographics for both groups and the type of patient care, location within the health system for the HCW were reported. Frequencies and percentages were calculated for categorical variables and means ± SD for continuous variables. The initial prevalence of cases in each group and seroconversions for the longitudinal follow-up of HCW for 2- and 6-month time points (i.e., the incidence) were reported. A multivariable logistic regression model was constructed to evaluate predictors of seroconversion rates during the 6-month interval, adjusted for age, gender, race and ethnicity, and co-habitation with a COVID-19 patient. HCW who had participated in a vaccine study and had positive serology at 6 months were excluded from the 6-month analysis.

### Publicly available COVID-19 dataset for the commonwealth of Virginia

In addition to the two datasets on seropositivity for HCW and community participants, we considered publicly available cumulative COVID-19 incident case rate data published weekly at the ZIP code level by the Virginia Department of Health. This dataset was densely observed across all ZIP codes in northern Virginia and is thus used to investigate the nature of spatial correlation in COVID outcomes, which informed the characterization of spatial random effects in the HCW seropositivity spatio-temporal mixed effect model introduced in what follows.

### Spatial correlation analysis

In our analyses, the geographical data considered are domiciliary ZIP codes for community participants, domiciliary ZIP codes for HCW, and ZIP codes from publicly available COVID-19 incident case rate data published at the ZIP code level by the Virginia Department of Health. To calculate distances for the spatial autocorrelation analysis and spatio-temporal mixed effects model, we compute the Euclidean distances between ZIP code centers characterized by their latitudes and longitudes. In the manuscript, these distances are expressed in miles using the Haversine formula for ease of interpretation [11]. To assess the impact of domiciliary location on serology positivity, which is commonly utilized in epidemiologic studies [12], the COVID-19 incident case data for viral detection from Virginia Department of Health (molecular and antigen testing) is used to assess the level of spatial correlation among neighboring ZIP codes. Among many measures of spatial association, Moran's I is one of the most widely used [13,14], which is utilized in this study. Given a set of observations of a variable of interest, $x_{1},x_{2},\ldots ,x_{N}$, and a measure of the distance between any two observations, $d_{ij}$ for i=1,2,…,Nand j=1,2,…,N, Moran's I measures the linear association between observations and neighboring observations weighted according to their respective distances such that:

$$I=\frac{N}{\sum_{i=1}^{N}\sum_{j=1}^{N}w\left(d_{ij}\right)}\frac{\sum_{i=1}^{N}\sum_{j=1}^{N}w\left(d_{ij}\right)\left(x_{i}-\bar{x}\right)\left(x_{j}-\bar{x}\right)}{\sum_{i=1}^{N}{\left(x_{i}-\bar{x}\right)}^{2}}$$

where $\bar{x}$ is the average of the N observations and w(·) is a weight function. For example, let $d_{ij}$ be the typical Euclidean distance. The inverse of the Euclidean distance can then be used to assign weights to observations such that $w\left(d_{ij}\right)=d_{ij}^{-1}$ for i≠j and $w(d_{ij})=0$ for i=j. However, in this study, we use a localized weight function such that $w_{k}\left(d_{ij}\right)=1$ for $d_{ij}\in \left(k-\alpha ,k+\alpha \right)$ and $w_{k}\left(d_{ij}\right)=0$ otherwise for a given distance interval midpoint k and interval half-width α. This distance-dependent weight function yields a localized Moran's I that can be used to assess the significance of the spatial correlation at various distances. Moran's I can take on values between −1 and +1 with positive values indicating positive linear association among neighboring observations.

### Spatio-temporal mixed effects modelling

In order to accurately estimate HCW seropositivity over the 6 months follow-up period, a spatio-temporal mixed effects model is developed that includes fixed effects for time, age, gender, race and ethnicity, type of patient care, and hospital location, as well as a spatial random effect to capture spatial correlation among observations. More specifically,

$$log\frac{p_{ij}}{1-p_{ij}}=\;\beta_{0}\;+\;\sum_{k=1}^{p}\beta_{k}X_{ijk}\;+\;t_{j}\;+\;S_{i}\left(v,\rho \right)\;+\;\in_{ij}$$

where $p_{ij}$ is the probability of seroprevalence for the ith HCW (i=1,…,N) at the jth time point (j=1,2,3), $X_{ijk}$ represents the observed value of the kth independent variable from the ith HCW at the jth time point, $\beta_{0}$ and $\beta_{1},\beta_{2},\ldots ,\beta_{p}$ are intercept and fixed effect coefficients respectively, $t_{j}$ is a fixed effect for the jth time point, $s_{i}\left(\nu ,\rho \right)$ is a spatial random effect, and $\in_{ij}$ is an independent, zero-mean error term. $s_{i}\left(\nu ,\rho \right)$ follows an N-dimensional zero mean normal distribution with correlation matrix R(ν,ρ) that characterizes the spatial autocorrelation among HCW, and $\in_{ij}$ follows a normal distribution $N(0,\sigma^{2})$.

The widely adopted Matérn correlation function [15,16] is used to characterize the spatial correlation such that ${\left[R(\nu ,\rho )\right]}_{ij}=\frac{2^{1-\nu }}{\Gamma (\nu )}{\left(\rho d_{ij}\right)}^{\nu }\;K_{\nu }\left(\rho d_{ij}\right)\;\text{where}d_{ij}$ is the distance between the domiciliary ZIP codes for the ith and jth HCW, ν is a smoothness parameter, ρ is a scale parameter, and $K_{\nu }\left(\cdot \right)$ is the modified Bessel function of the second kind of order ν. Setting ν=0.5 results in an exponential correlation function ${\left[R(\nu ,\rho )\right]}_{ij}=exp\left(-\rho d_{ij}\right)$ for which the rate of decay in the spatial correlation as distance increases is controlled by ρ. ρ is then set such that the spatial correlation by distance for this model is similar to the spatial correlation by distance in the COVID-19 incident case data. The Akaike Information Criterion is used to evaluate model fit and select the most parsimonious model[17]. Data analyses were conducted using R statistical software (v.4.0.3; R Foundation for Statistical Computing, Vienna, Austria). The institutional review board at Inova Health and University of Virginia approved this study.

### The role of funding source

This original research study was funded by a seed grant from the Inova Health System to support the health and wellbeing of HCWs in the work environment during the pandemic.

## Results

### Demographic characteristics and seropositivity

Of a total of 1819 asymptomatic HCW enrolled in this prospective cohort study, 1473 (81%) had serology and clinical data at the two-months interval, and 1323 (73%) of the participants had data at 6-months interval. Of those with follow-up data, the majority were <50 years of age (73.4%), more likely to be women (*p* <0.001), and belong to White non-Hispanic ethnic groups (*p* = 0.002). Most participants had direct patient contact and 70% were enrolled from the tertiary referral hospital (**Supplemental Table 1**). At six months, 27% of the study population were no longer working at the health system or declined to participate and characteristics of these patients are presented in **Supplementary Table 2**.

At baseline (April/May 2020), 21 (1.2%) HCW were found to have positive SARS-COV-2 serology. The highest prevalence of positive serology was among young HCW less than 30 years of age (prevalence = 2.6%) and Black HCW (prevalence = 4.1%). There was no appreciable difference in the prevalence of COVID-19 disease by the type of patient care or hospital location (Table 1). At two months interval, the incidence rate of COVID-19 positive serology was 2.8%. In June/July 2020 (i.e., two-month time point), when clinical COVID-19 testing was more readily available, 29/41 (70.7%) of seropositive HCW reported having a positive COVID-19 test and of the 33 HCW who reported having a positive COVID-19 test, 3/33 (9.1%) failed to seroconvert. Similar to the trends at baseline, participants who belong to the 20–29 age category had the highest positive serology incidence rate (5.6%). By six months interval, the overall incidence increased to 4.8%. Participants 20–29 years of age and those of Black race had a higher incidence rate than other participants, but rates were similar according to other characteristics including gender, direct versus indirect patient care, and location of the hospital (Table 1). In univariable and multivariable models evaluating the factors associated with positive serology at six months, the odds of COVID-19 positive serology were the highest among HCW who belong to the youngest age group, Black race, and those with exposure to COVID-19 at home. Home exposure of COVID-19 has a large impact on HCW positive serology outcomes. The odds of getting a new positive serology result in workers who have been exposed to COVID-19 at home is thirteen times as high as those without a home exposure (**Supplemental Fig. 1**).

**Table 1:** Cross-sectional seropositivity rates at each time point (period prevalence).

Variables	Baseline	Two Months	Six Months
Overall	21/1819 (1.2%)	41/1473 (2.8%)	64/1323 (4.8%)
Age by decade, years, n (%)			
20–29	10/381 (2.6%)	17/302 (5.6%)	20/253 (7.9%)
30–39	5/543 (0.9%)	11/436 (2.5%)	18/381 (4.7%)
40–49	3/424 (0.7%)	9/340 (2.6%)	12/321 (3.7%)
50–59	1/310 (0.3%)	2/257 (0.8%)	9/238 (3.8%)
≥60	2/161 (1.2%)	2/138 (1.4%)	5/130 (3.8%)
Gender, n (%)			
Female	18/1432 (1.3%)	35/1196 (2.9%)	49/1070 (4.6%)
Male	3/387 (0.8%)	6/277 (2.2%)	15/253 (5.9%)
Race & Ethnicity, n (%)			
White	7/1031 (0.7%)	22/856 (2.6%)	31/775 (4.0%)
Black	9/219 (4.1%)	5/168 (3.0%)	13/147 (8.8%)
Hispanic	2/165 (1.2%)	4/122 (3.3%)	8/104 (7.7%)
Other	3/404 (0.7%)	10/327 (3.1%)	12/297 (4.0%)
Type of Patient Care, n (%)			
Direct	19/1427 (1.3%)	36/1171 (3.1%)	51/1051 (4.9%)
Non-direct	2/392 (0.5%)	5/302 (1.7%)	13/272 (4.8%)
Location, n (%)			
Inova Fairfax	18/1317 (1.4%)	33/1034 (3.2%)	43/948 (4.5%)
Other	3/502 (0.6%)	8/439 (1.8%)	21/375 (5.6%)

### Northern VA community: demographic characteristics and seropositivity

Similar to the HCW cohort, the majority of participants (*n* = 949) were less than 50 years of age, female participants, and they belong to the white non-Hispanic ethnic group (**Supplemental Table 1**). The overall incidence of COVID-19 in the community was 4.5%. The highest estimates were observed among the participants younger than 50 years of age or older than 70 years. Similar to HCW, the incidence among Black and Hispanic participants were higher than non-Hispanic Whites (**Supplemental Table 3**). After adjustment, non-White Hispanics were at the highest risk in the community (adjusted OR vs. non-Hispanic of 13.49 with 95% confidence interval (6.46, 30.10)).

### Northern VA community: spatial correlation in COVID-19 case rates

**Fig. 1:**
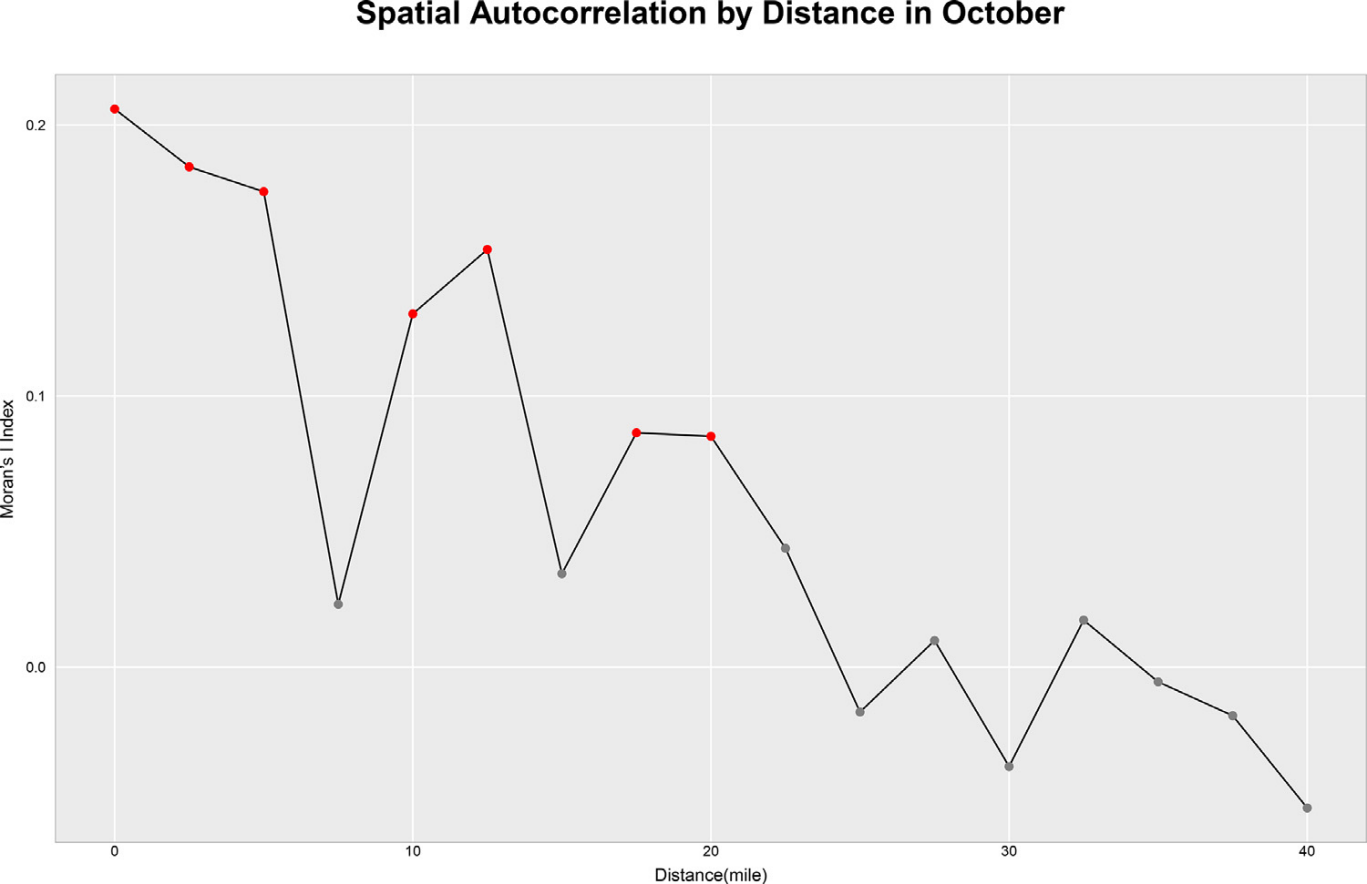
Spatial correlation by distance. Moran's I is a commonly used measure of spatial autocorrelation. This figure illustrates the correlation between COVID-19 case rates by zone improvement plan (ZIP) code and case rates from other ZIP codes at different distances (in miles) in October 2020. Distances exhibiting significant spatial correlation (p-value < 0.05) are indicated by red points. Spatial correlation decreases with distance, and locations exhibit significant correlation with other locations within a 20-mile radius.

Fig. 1 illustrates the correlation between community COVID-19 case rates by ZIP code and case rates from other ZIP codes at different distances (in miles) in October 2020, which corresponds to the 6-month interval. Each point represents the mid-point of a distance interval with a width of 5 miles. Distances exhibiting significant spatial correlation (*p-*value < 0.05) are indicated by red points. This figure reveals that spatial correlation decreases with distance, and locations exhibit significant correlation with other locations within a 20-mile radius. **Supplemental Fig. 2** illustrates the correlation between community COVID-19 case rates by ZIP code and case rates from other ZIP codes at different distances (in miles) for three time points: May 2020, June and July 2020, and October 2020.

### HCW: cross sectional and spatio-temporal models

Results from the cross-sectional multivariable logistic regression model for HCW at 6-month are shown in Table 2. COVID-19 positive serology was the highest among HCW between the ages of 20–29 years with significantly lower incidence of positive serology among HCW over 30 years of age (odds ratios ranging from 0.40 to 0.48). Black HCW exhibited significantly higher incidence of COVID-19 positive serology compared to white HCW (odds ratio of 2.53 with 95% confidence interval (1.20,5.05)). Exposure to COVID-19 at home was also associated with increased incidence of COVID-19 positive serology [odds ratio 13.77 with 95% confidence interval (5.92, 34.30)]. There was no difference in odds of seropositivity based on non-Hispanic versus Hispanic ethnicity among HCW. Furthermore, no significant impact on the incidence of positive COVID-19 serology at 6-month follow-up were found with respect to gender (adjusted OR male vs. female of 1.52 with 95% confidence interval (0.77, 2.77)), self-described job description with direct or not-direct patient contact (adjusted OR non-direct vs. direct of 0.93 with 95% confidence interval (0.45, 1.73)), and the hospital location within the healthcare system (adjusted OR other vs. Inova Fairfax of 1.32 with 95% confidence interval (0.73, 2.29))

**Table 2:** Cross-sectional model at 6-month estimation.

Variable	Crude Odds Ratio	OR 95% CI		Adjusted Odds Ratio	OR 95% CI	
		Lower Bound	Upper Bound		Lower Bound	Upper Bound
Decade of Life						
20–29	1.00	–	–	1.00	–	–
30–30	0.58	0.30	1.12	0.48	0.23	0.96
40–49	0.46	0.21	0.94	0.44	0.19	0.92
50–59	0.47	0.19	1.01	0.41	0.16	0.93
≥60	0.50	0.15	1.23	0.40	0.11	1.05
Gender						
Female	1.00	–	–	1.00	–	–
Male	1.34	0.70	2.36	1.52	0.77	2.77
Race & Ethnicity						
White	1.00	–	–	1.00	–	–
Black	2.37	1.15	4.51	2.53	1.20	5.05
Hispanic	2.08	0.84	4.38	1.37	0.50	3.09
Other	1.03	0.49	1.97	1.07	0.50	2.07
Type of Patient Care						
Direct	1.00	–	–	1.00	–	–
Non-direct	1.01	0.51	1.82	0.93	0.45	1.73
Location						
Inova Fairfax	1.00	–	–	1.00	–	–
Other	1.26	0.72	2.12	1.32	0.73	2.29
Home COVID-19 Exposure						
No	1.00	–	–	1.00	–	–
Yes	15.26	6.60	33.66	13.77	5.92	34.30

Abbreviations: CI = confidence interval; COVID-19 = Coronavirus Disease 2019; OR = odds ratio.

**Fig. 2:**
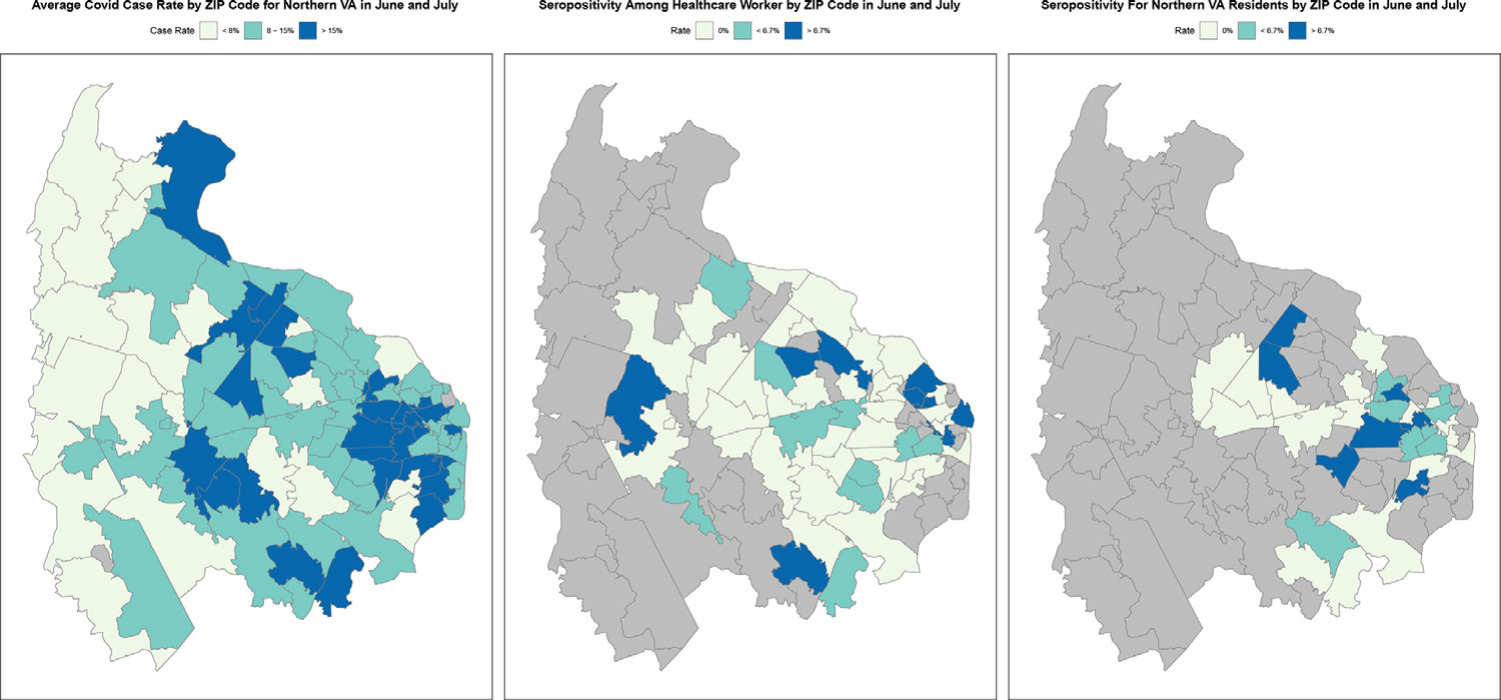
COVID-19 case rate by zone improvement plan (ZIP) code in Northern Virginia over June to July 2020: (A) Virginia Department of Health data on COVID-19 molecular and antigen testing confirmed rate; (B) seropositive rate of COVID-19 disease among healthcare workers; (C) seropositive COVID-19 rate of research participants from the community.

The average COVID-19 case rate in Northern VA as reported by the Virginia Department of Health, HCW seropositivity, and seropositivity in participants from the community by their ZIP code are presented in Fig. 2 from June/July 2020 when community serology data was available. In this figure, the zip codes with the highest seropositivity among HCW corresponds to intermediate to high seropositivity rates in the community and Virginia Department Health data. In fact, both HCW seropositivity and seropositivity in participants from the community exhibit significant spatial cross-correlation with average COVID-19 case rates in Northern VA (*p*-values 0.006 and <0.001 respectively) [18].

Evidence of spatial correlation in the community COVID-19 case rates motivate the development of a spatio-temporal mixed effects model for HCW (Table 3). Since significant spatial correlation in community COVID-19 case rates is noted among locations with distances of 20 miles or less, the scale parameter for the spatial correlation function, ρ, is set to 8.33 so that the spatial correlation function for this model also exhibits significant autocorrelation for distances of 20 miles or less (**Supplemental Fig. 3)**. The results show that higher odds ratios for positive test outcome were found at 2- and 6- month time points, 4.3 times and 6.9 times higher than baseline. Results of other variables in the mixed effect model align with the cross-sectional model. Interaction effects between time and each variable were considered, but only the interaction between time and race and ethnicity produced a significant interaction effect with the 2-month time point. Overall, the odds ratio for Black HCW is higher than White non-Hispanic workers, which aligns with the cross-sectional model. The Akaike Information Criterion (AIC) values for successive model fits obtained by adding each variable one at a time (**Supplemental Fig. 4**) suggest that accounting for time, age, race and ethnicity, and spatial correlation improved the model fit, as noted by the reduction in the AIC values, while adding other factors did not improve the model fit.

**Table 3:** Spatial-temporal mixed effect model estimation.

Variable	Crude Odds Ratio	OR 95% CI		Adjusted Odds Ratio	OR 95% CI	
		Lower Bound	Upper Bound		Lower Bound	Upper Bound
Time						
Baseline	1			1		
2-Months	4.20	1.82	11.07	4.32	1.86	11.41
6-Months	6.61	2.96	17.09	6.88	3.06	17.85
Decade of Life						
20–29	1			1		
30–30	0.45	0.27	0.74	0.43	0.26	0.72
40–49	0.44	0.25	0.76	0.42	0.23	0.76
50–59	0.28	0.13	0.56	0.28	0.13	0.57
60+	0.56	0.24	1.20	0.61	0.25	1.36
Gender						
Female	1			1		
Male	0.91	0.54	1.47	1.07	0.61	1.78
Race & Ethnicity						
White	1			1		
Black	5.19	1.74	16.07	6.45	2.13	20.32
Hispanic	2.01	0.29	8.88	1.72	0.24	7.82
Other	1.30	0.27	4.92	1.43	0.30	5.45
Type of Patient Care						
Direct	1			1		
Non-direct	0.71	0.40	1.18	0.70	0.39	1.20
Location						
Inova Fairfax	1			1		
Other	0.65	0.40	1.02	0.72	0.44	1.16
Interaction Terms†						
2-Months x Black	0.15	0.03	0.64	0.15	0.03	0.63
6-Months x Black	0.33	0.09	1.18	0.32	0.08	1.15
2-Months x Hispanic	0.73	0.11	6.32	0.77	0.11	6.79
6-Months x Hispanic	1.11	0.20	8.81	1.18	0.20	9.58
2-Months x Other	1.06	0.23	5.89	1.04	0.22	5.79
6-Months x Other	0.95	0.21	5.16	0.90	0.20	4.91

Abbreviations: CI = confidence interval; OR = odds ratio.

† Reference group: Time: baseline; Race and Ethnicity: White.

## Discussion

This is a prospective serology study of (1) asymptomatic HCW in a large regional healthcare system and (2) asymptomatic participants from the same communities in Northern Virginia. The major findings of this study are as follows: (1) The overall prevalence of COVID-19 exposure reflected by serology testing in HCW at baseline in April to May 2020 near the beginning of the pandemic was low; (2) with implementation of public health measures and PPE in hospital settings the overall incidence rate of SARS-CoV-2 exposure reflected by incident serology among HCW over the next 6 months of the pandemic remained low (~ 4.8%); (3) the incidence rate of COVID-19 disease among HCW is similar to the incidence rate of the disease among asymptomatic participants from the community with the important exception of Hispanic ethnicity potentially reflecting different socio-economic factors among those Hispanics living in the community and the subset employed in the regional healthcare system; (4) the main factors that influence the incidence rate among HCW are younger age groups, race, residential zip code, and exposure to a COVID-19 infected individual within the household and not factors such as direct patient contact or work location within the healthcare system (**Fig. 4**).

The COVID-19 disease has exerted a heavy toll on HCW including physicians, nursing staff, and allied health professional since the beginning of the pandemic. Despite the significant impact of the disease on healthcare systems at large, HCW have shown remarkable resilience in providing care during the pandemic[19]. Early during the pandemic, the prevalence of COVID-19 among asymptomatic healthcare workers was quite low (~prevalence 0 to 1.6%), which is similar to the point prevalence in our study (~1.2%) [20,21]. However, as time has progressed, the exposure of HCW to the disease has become more burdensome, especially among those with direct contact with COVID-19 patients. Shah et al. evaluated the risk of transmission of COVID-19 among HCW in patient and non-patient facing roles[22]. They found that HCW and their households accounted for 17.2% of the total COVID-19 admission in Scotland, while representing only 11.2% of the population[22]. In the United States, Erdem et al. examined COVID-19 infection among HCW and estimated that there are more than 114,529 infections among HCW representing 34 cases per 100,000 individuals among the U.S. population [23].

As part of a systematic effort to screen and limit the COVID-19 infection among HCW, serial serologic-based testing has been suggested for use in the population as a whole to (1) understand the COVID-19 epidemiology; (2) assess an individual's previous SARS-CoV-2 exposure, and neutralization antibody [2]. Using serologic testing, the prevalence of COVID-19 among asymptomatic HCW in our study at baseline was quite low (~1.2%) and they remained low at 2 and 6-months follow-up (~4.8%). In a longitudinal study of both symptomatic and asymptomatic HCW in the U.K., the prevalence of seropositive result was much higher (9.4%) [24]. Nguyen et al. performed a prospective cohort study of the general community including HCW and found that of the 99,795 frontline HCW included, only 5545 (5.5%) had positive serology, which is consistent with our estimates [25]. However, unlike the results of that study, the incidence rates at 2 and 6-months estimates in our study were similar to that in the general community. The peak of the number of PCR positive COVID-19 cases among HCWs corresponded to that in the community at large, which is consistent with our results.

**Fig. 3:**
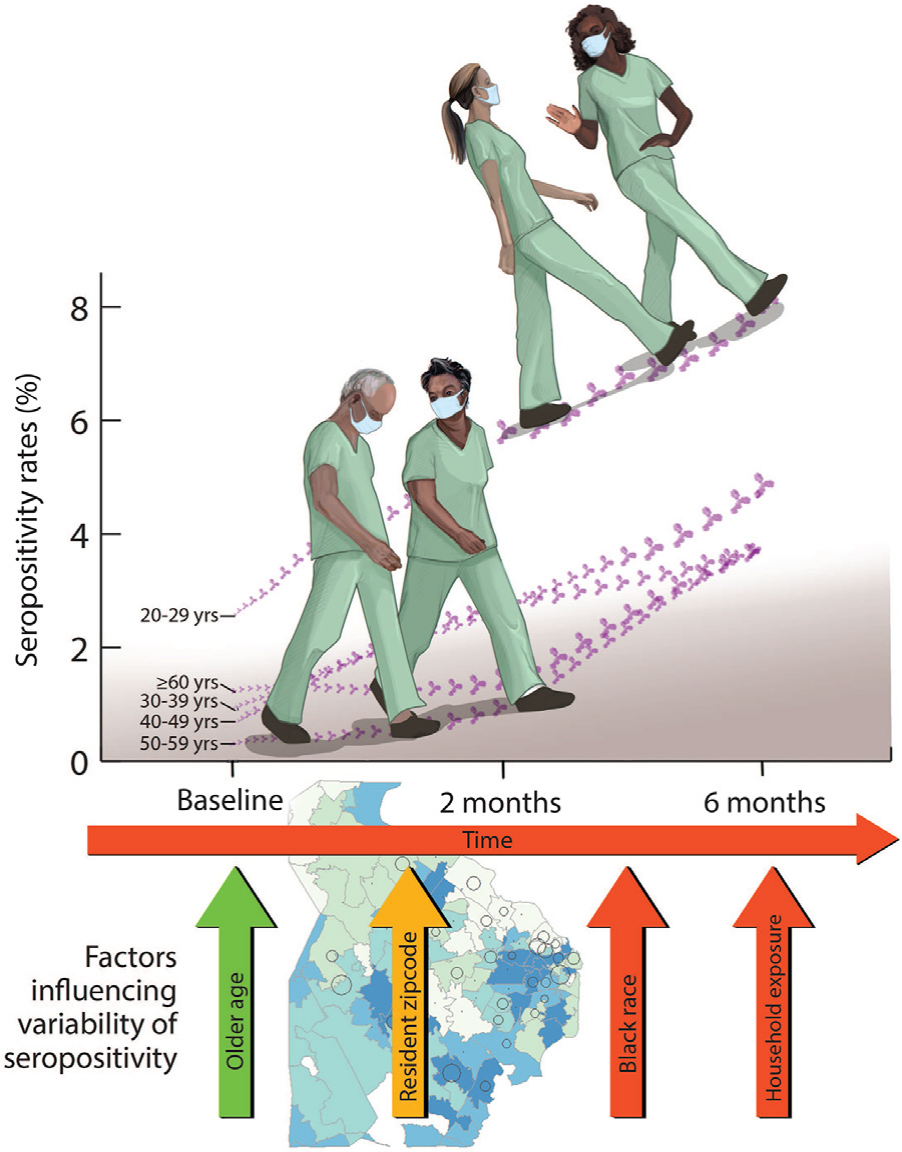
(Central Illustration): Factors considered to influence the seropositive rates of COVID-19 disease among healthcare workers in Northern Virginia in longitudinal model over six months. Red arrows were significant factors that increased risk, yellow arrow associated with increased and decreased risk, and green arrow represents decreased risk for seroconversion in a multivariate model.

Several reasons can explain the similar seropositive rate among HCW and among participants living in the same community. First, the strict implementation of the public health measures suggested by the Centers of Disease Control and Prevention in healthcare systems had a substantial effect on healthcare delivery and access to care. In Northern Virginia, the use of gowns, gloves, handing washing, eye protection, and several layers of personal protective devices became mandatory early April as the cases were rising. Second, the cancelation of elective procedures and surgeries, the utilization of telemedicine, and development of specialized COVID units played an important role in limiting the transmission rates [7]. At six-months follow-up, the factors that influenced the transmission of new SARS-CoV-2 infection include younger age group, African American ethnicity, residency and ZIP-code, and contact with a COVID-19 + household member (Fig. 3). In a living document that systematically collects data on the epidemiology and risk factors for COVID-19 disease in HCW, ethnic minority as a class, including Black race, was associated with increased risk of infection, which is consistent with our findings [4]. Further, our results show that the strength of the spatial correlation among nearby locations (as measured by Moran's I) decreases over time (**Supplemental Fig. 2)**, which may be associated with the adoption of public health measures designed to limit COVID-19 exposure within the community. Factors specific to the healthcare system including direct vs. indirect exposure to COVID-19 unit, the location of the hospital within the system did not have a significant effect on the infection among HCWs. This is consistent with a prior observation by Steenssels and colleagues, who reported that neither being directly involved in COVID-19 care or nor working in COVID-19 unit increased the odds of seropositive infection, but home exposure was also a major factor associated with positive serology [26].

This study is not without limitations. First, of the initial 1819 patients enrolled, 1473 (81%) had serologic data during two-months interval, and 1323 (73%) had serologic data at 6-months follow-up. The highest dropout rates occurred among adults age 20–29 and 30–39 years. Participants who belong to ethnic minorities also showed higher dropout rates. Because of the impact of the pandemic, many allied health professionals (disproportionately represented by underrepresented minorities) left the healthcare system, and they were not willing to continue to participate in a healthcare study despite efforts by the investigators to engage their interest. However, given that this large study started early during the pandemic, at a time of high anxiety the majority of the study population agreed to participate and be followed up despite their demanding schedules. Second, there is the potential of self-referral by HCW including physicians, nursing staff, and allied healthcare providers to monitor their seropositive rates as they care for COVID-19 patients. To mitigate this effect, the investigators provided open access to any healthcare provider or allied professional to be enrolled in the study with multiple enrolment sites in close proximity within the healthcare system to where they worked or congregated (i.e. near break rooms) and daily appointments during the individual's follow-up period ± seven days. Throughout the period of the study, advertising and marketing throughout the healthcare system via electronic and written communications, and townhall meetings were implemented to increase awareness on this systematic effort to enrol and retain healthcare participants. Third, the Ortho-Clinical Diagnostics VITROS “Anti-SARS-CoV-2 Total Reagent Pack” is currently reported to have a specificity of 100% (400/400) with a 95% CI (99–100%) [9]. A specificity of 99.5% would be realistic suggesting approximately 9 initial false-positive tests given our sample size. However, in a prior publication evaluating a viral neutralization assay in the baseline positive binding assay cohort the presence of neutralization was seen in all tested participants samples [2,27]. Fourth, we did not perform an orthogonal serologic testing strategy for population surveillance because the serologic testing used in this study had very high specificity and negative predictive value [2]. Fifth, some variables in this study are self-identified or self-described, which can potentially introduce response bias. Finally, the use of two separate assays targeting different antibodies to SARS-CoV-2 may limit direct comparisons between HCWs and the community, but generally given the very high sensitivity and specificity for both assays they can give an estimate on seropositivity between groups.

## Conclusion

In Northern Virginia, the seropositive rate of COVID-19 disease among HCW was comparable to that in the community. Future studies are needed to evaluate which factors implemented in the health system were most effective at reducing risk to be similar to the surrounding community despite frequent exposure to COVID-19 infected patients.

## Appendix Supplementary materials

Image, application 1

Image, image 2

Image, image 3

Image, image 4

Image, image 5
