# Supplementary material for: Seropositivity of COVID-19 among asymptomatic healthcare workers: A multi-site prospective cohort study from Northern Virginia, United States
Source: Lancet Reg Health Am. 2021 Jul 29;2:100030. doi: 10.1016/j.lana.2021.100030 (PMC8319689; doi:10.1016/j.lana.2021.100030)
Supplement: Supplementary file 1 [file mmc1.docx]

**Supplementary Table 1.** Baseline characteristics of the asymptomatic study populations in Northern Virginia; Healthcare workers at baseline and 2-months interval and community sample from Northern Virginia.

| **Variables** | **Healthcare Workers†**  **Baseline (April/May 2020)** | **Healthcare Workers^†^**  **2 months (June/July 2020)**  **(n=1,473)** | **Northern VA Community^‡^**  **July 2020** |
| --- | --- | --- | --- |
|  | **(n=1,819)** |  | **(n=949)** |
| **Age Category, %** |  |  |  |
| 20-29 | 381/1819 (20.9%) | 302/1473 (20.5%) | 166/949 (17.5%) |
| 30-39 | 543/1819 (29.9%) | 436/1473 (29.6%) | 206/949 (21.7%) |
| 40-49 | 424/1819 (23.3%) | 340/1473 (23.1%) | 184/949 (19.4%) |
| 50-59 | 310/1819 (17.0%) | 257/1473 (17.4%) | 170/949 (17.9%) |
| ≥60 | 161/1819 (8.9%) | 138/1473 (9.4%) | 223/949 (23.5%) |
| **Gender** |  |  |  |
| Male | 387/1819 (21.3%) | 277/1473 (18.8%) | 353/949 (37.2%) |
| Female | 1432/1819 (78.7%) | 1196/1473 (81.2%) | 596/949 (62.8%) |
| **Race** |  |  |  |
| White | 1031/1819 (56.7%) | 856/1473 (58.1%) | 528/949 (55.6%) |
| Black | 219/1819 (12.0%) | 168/1473 (11.4%) | 126/949 (13.3%) |
| Other | 569/1819 (31.3%) | 449/1473 (30.5%) | 295/949 (31.1%) |
| **Ethnicity** |  |  |  |
| Not Hispanic | 1654/1819 (90.9%) | 1351/1473 (91.7%) | 773/949 (81.5%) |
| Hispanic | 165/1819 (9.1%) | 122/1473 (8.3%) | 176/949 (18.6%) |
| **Type of Patient Care** |  |  |  |
| Direct | 1427/1819 (78.4%) | 1171/1473 (79.5%) | - |
| Non-Direct | 392/1819 (21.6%) | 302/1473 (20.5%) | - |
| **Location** |  |  |  |
| Inova Fairfax | 1317/1819 (72.4%) | 1034/1473 (70.2%) | - |
| Other | 502/1819 (27.6%) | 439/1473 (29.8%) | - |

^†^ Data are presented as proportions and percentages of total sample size.

^‡^ Two months HCW data was collected contemporaneously to Northern VA community data

**Supplementary Table 2.** Comparison of 6-months dropout rates according to baseline characteristics.

| **Variables** | **Dropout Rate** | ***P*-value** |
| --- | --- | --- |
| **Overall** | 499/1810 (27.4%) |  |
| **Age by decade, n (%)** |  | 0.002 |
| 20-29 | 128/381 (33.6%) |  |
| 30-39 | 162/543 (29.8%) |  |
| 40-49 | 104/425 (24.5%) |  |
| 50-59 | 73/311 (23.5%) |  |
| ≥60 | 32/162 (19.8%) |  |
| **Gender, n (%)** |  | <0.001 |
| Female | 365/1435 (25.4%) |  |
| Male | 134/387 (34.6%) |  |
| **Race & Ethnicity, n (%)** |  | 0.002 |
| White | 258/1033 (25.0%) |  |
| Black | 73/220 (33.2%) |  |
| Hispanic | 61/165 (37.0%) |  |
| Other | 107/404 (26.5%) |  |
| **Type of Patient Care, n (%)** |  | 0.097 |
| Direct | 378/1429 (26.5%) |  |
| Non-direct | 121/393 (30.8%) |  |
| **Location, n (%)** |  | 0.265 |
| Inova Fairfax | 371/1319 (28.1%) |  |
| Other | 128/503 (25.4%) |  |

**Supplementary Table 3.** Positive SARS CoV-2 serology incidence and adjusted risk for the Northern Virginia community without known COVID-19 at baseline.

|  | Incidence (%) | Unadjusted OR | Adjusted OR |
| --- | --- | --- | --- |
| Overall | 43/949 (4.53 %) |  |  |
| Age by decade |  |  |  |
| 18-29 years | 9/166(5.42%) | 1.0 | 1.0 |
| 30-39 years | 11/206 (5.33%) | 0.98 (0.40, 2.50) | 0.84 (0.32, 2.25) |
| 40-49 years | 12/184 (6.52%) | 1.22 (0.50, 3.06) | 0.97 (0.38, 2.58) |
| 50-59 years | 4/170 (2.35%) | 0.42 (0.11, 1.32) | 0.38 (0.10, 1.27) |
| 60-69 years | 2/131(1.53%) | 0.27 (0.04, 1.07) | 0.10 (0.01, 0.59) |
| ≥70 years | 5/92 (5.43%) | 1.00 (0.30, 3.00) | 1.25 (0.35, 4.07) |
| Gender |  |  |  |
| Female | 24/596 (4.03%) | 1.0 | 1.0 |
| Male | 18/350 (5.14%) | 1.29 (0.68, 2.41) | 1.75 (0.87, 3.48) |
| Race |  |  |  |
| White | 21/528 (3.98%) | 1.0 | 1.0 |
| Black | 6/126 (4.76%) | 1.21 (0.43, 2.89) | 2.94 (0.96, 8.27) |
| Other | 16/295 (5.42%) | 1.38 (0.70, 2.69) | 0.89 (0.42, 1.83) |
| Ethnicity |  |  |  |
| Not Hispanic | 16/773 (2.07%) | 1.0 | 1.0 |
| Hispanic | 27/176 (15.34%) | 8.57 (4.56, 16.64) | 13.49 (6.46, 30.10) |
