# Supplementary figures and images for: Seropositivity of COVID-19 among asymptomatic healthcare workers: A multi-site prospective cohort study from Northern Virginia, United States
Source: Lancet Reg Health Am. 2021 Jul 29;2:100030. doi: 10.1016/j.lana.2021.100030 (PMC8319689; doi:10.1016/j.lana.2021.100030)

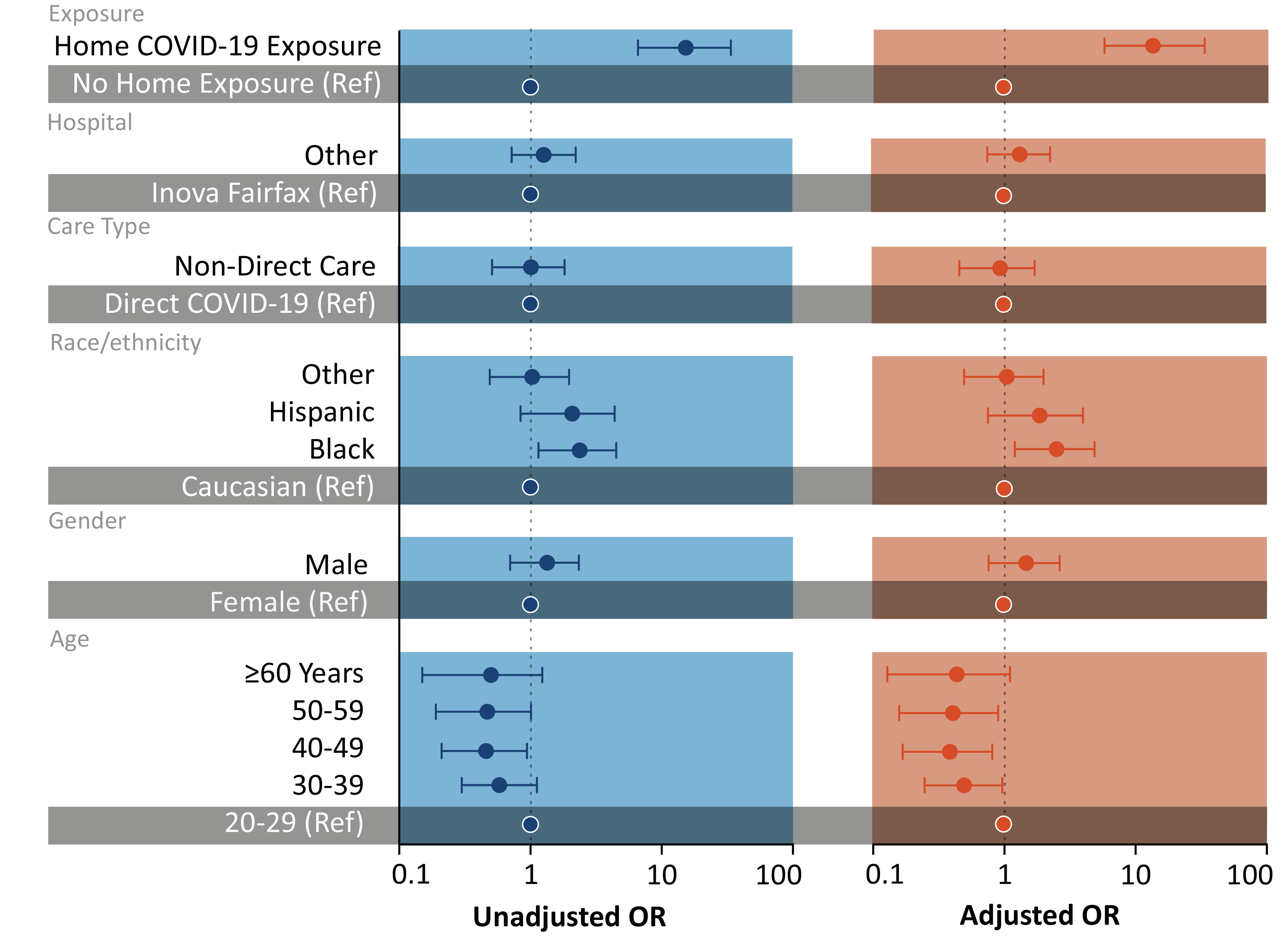

Supplement: Supplementary file 2 [file mmc2.jpg]

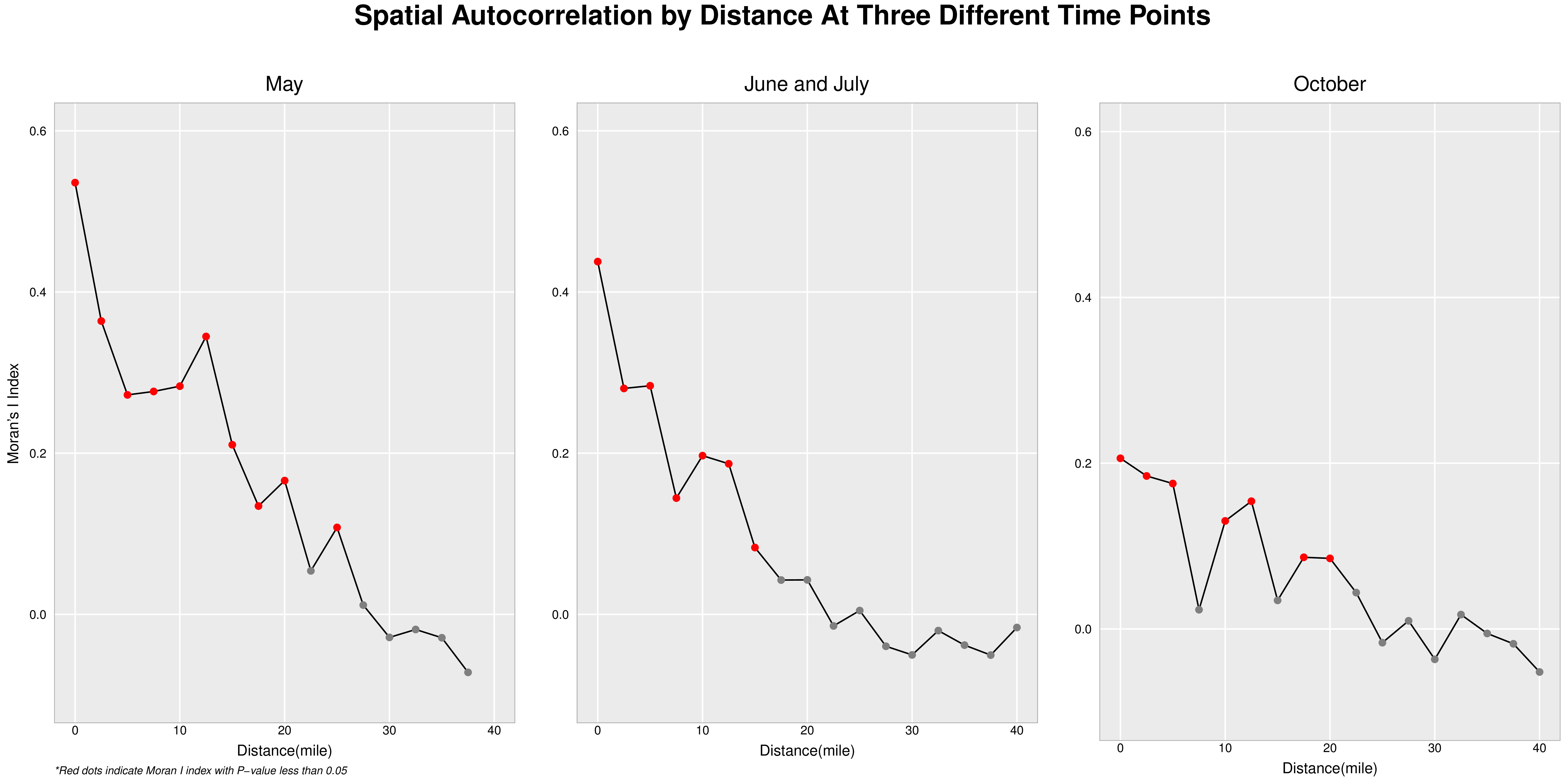

Supplement: Supplementary file 3 [file mmc3.jpg]

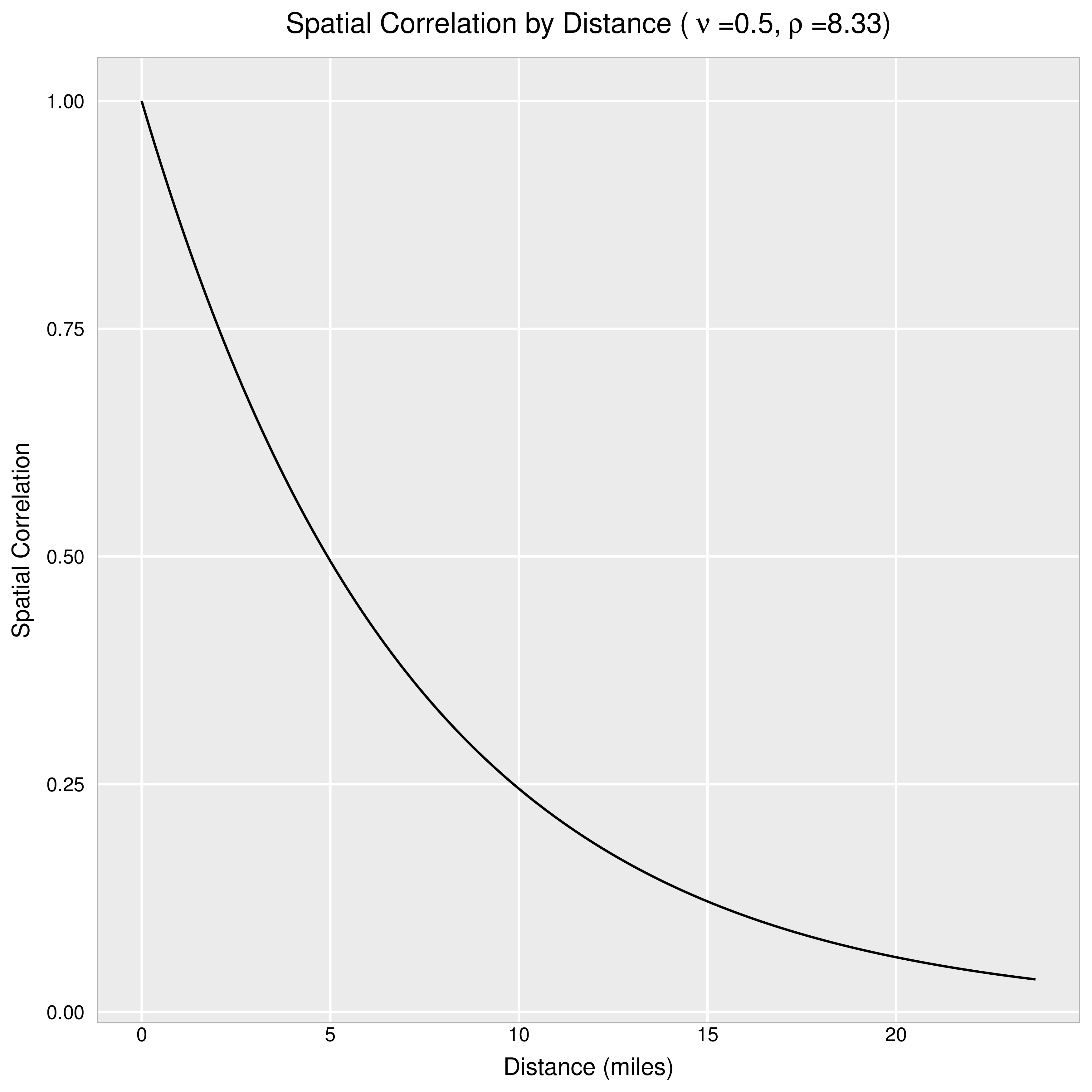

Supplement: Supplementary file 4 [file mmc4.jpg]

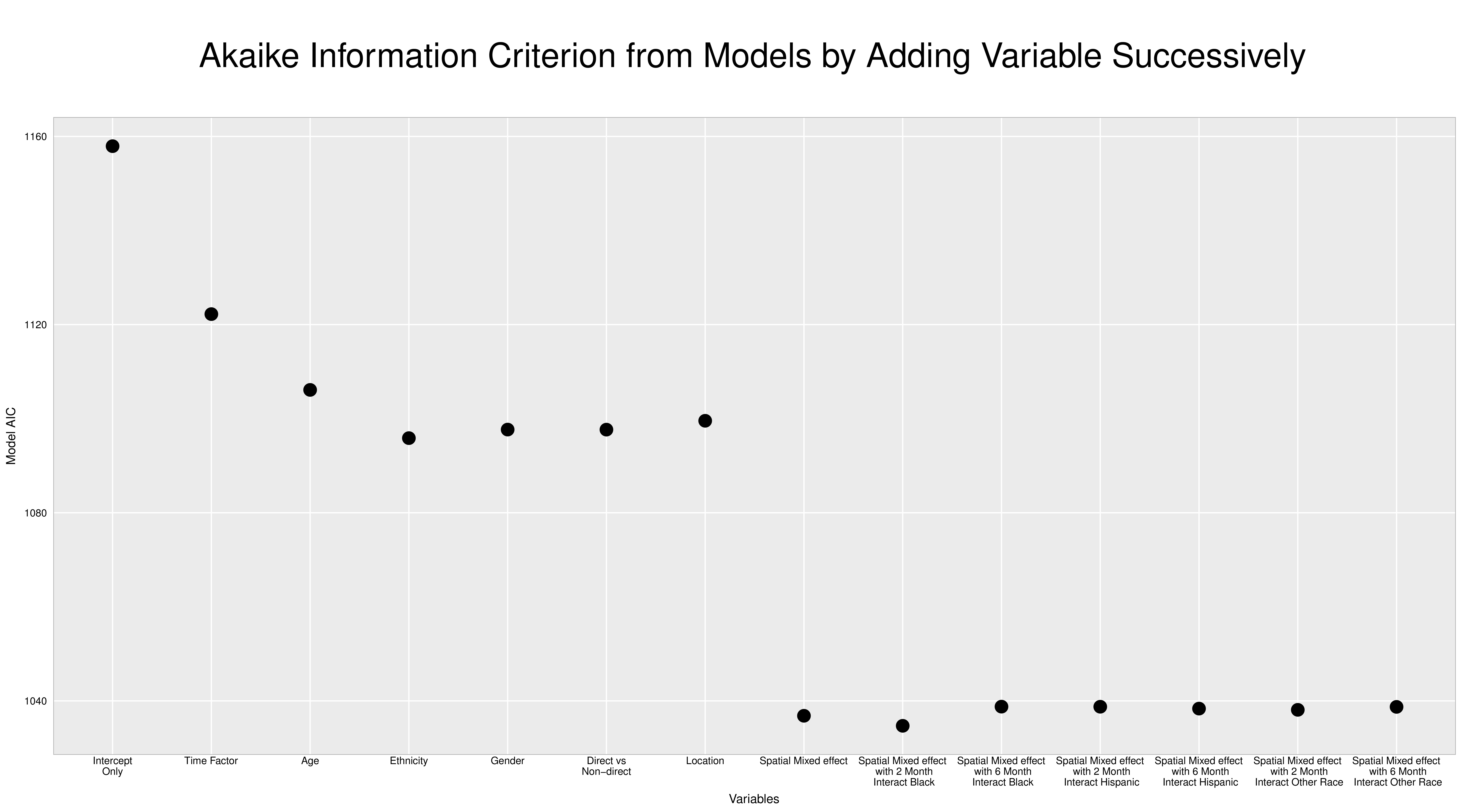

Supplement: Supplementary file 5 [file mmc5.jpg]
